# Arterial leg ulcers—Bacterial patterns, antimicrobial resistance and clinical characteristics, a retrospective single-centre cohort, 2012–2021

**DOI:** 10.1371/journal.pone.0290103

**Published:** 2023-08-11

**Authors:** Jonas Salm, Tanja Böhme, Elias Noory, Ulrich Beschorner, Tobias Siegfried Kramer, Dirk Westermann, Thomas Zeller

**Affiliations:** 1 Department of Cardiology and Angiology, University Heart Center Freiburg—Bad Krozingen, Medical Center–University of Freiburg, Faculty of Medicine, University of Freiburg, Freiburg im Breisgau, Germany; 2 Charité –Universitätsmedizin Berlin, Institute for Hygiene and Environmental Medicine, Corporate Member of Freie Universität Berlin and Humboldt-Universität zu Berlin, Berlin, Germany; 3 LADR der Laborverbund Dr. Kramer & Kollegen, Geesthacht, Germany; Defense Threat Reduction Agency, UNITED STATES

## Abstract

**Objective:**

Severe wound infections in patients with peripheral artery disease (PAD) are common, potentially life- and limb-threatening, and difficult to treat. Evidence on patients with infected leg ulcers in PAD is scarce. This study aims to provide insight into the microbiological patterns and antimicrobial resistance (AMR) of specific pathogens in patients with arterial leg ulcers.

**Methods and design:**

In this retrospective, consecutive, single-centre study 16,553 patients underwent an endovascular revascularization procedure between 2012 and 2021. Of these, 1,142 patients had PAD Rutherford category 5 or 6 with infected leg ulcers. Logistic regression was used to identify risk factors for *Staphylococcus aureus*-associated infections.

**Results:**

A total of 3,431 bacterial isolates were detected, of which 2,335 (68.1%) bacterial isolates were gram-positive and 1,096 (31.9%) were gram-negative species. The most prevalent bacteria were *S*. *aureus* (18.6%), *Enterococcus faecalis* (9.1%) and *S*. *epidermidis* (7.8%). *Pseudomonas aeruginosa* (5.6%), *Proteus mirabilis* (3.7%) and *Escherichia coli* (3.4%). The resistance of *S*. *aureus* isolates to clindamycin was 11.0%. Resistance to oxacillin was rare (1.5%). *P*. *aeruginosa* is frequently resistant to ciprofloxacin (14.4%) whilst intrinsically resistant to trimethoprim/sulfamethoxazole. *P*. *mirabilis* and *E*. *coli* were frequently resistant to both ciprofloxacin (7.3; 20.7%) and trimethoprim/sulfamethoxazole (24.6; 22.6%), respectively. Resistance to amoxicillin/clavulanic acid was high among *E*. *coli* isolates (36.8%). Multi-drug resistance (MDR) was rare among *S*. *aureus* and *P*. *aeruginosa* isolates. In contrast, the proportion of MDR was high in *E*. *coli* isolates. End-stage renal disease was independently positively associated with *S*. *aureus* identification (p = .042).

**Conclusion:**

*S*. *aureus* was the most common pathogen in arterial leg ulcers with end-stage renal disease being an independent risk factor. Clindamycin resistance was common, making empirical therapy likely to fail. Isolated *E*. *coli* species had a high proportion of MDR.

## Introduction

Peripheral artery disease (PAD) affects approximately 200 million people worldwide [1]. It is associated with a threefold increase in mortality compared with people without PAD [2]. The excess risk of death in people with PAD is partly explained by a 6-fold higher relative risk of death from cardiovascular disease, such as coronary heart disease, compared with people without evidence of PAD [2]. However, a subanalysis of the EUCLID trial (a randomised, double-blind trial of ticagrelor versus clopidogrel in people with symptomatic PAD) shows that cardiovascular causes of death were only slightly more common in people with PAD (55.9%) than non-cardiovascular causes (41.3%) [3]. The most common non-cardiovascular causes of death were malignancy (17.9%) and infection (11.9%) [3]. Looking only at PAD patients with chronic limb-threatening ischemia (CLTI), the risk of death from infection/sepsis is increased compared to PAD patients without CLTI (17.6% vs. 10.9%).

PAD is clinically classified according to Rutherford categories. This classification is based on the severity of symptoms and ranges from stage 1 to 6 with increasing severity of the disease. Patients with leg ulcers are classified as Rutherford category 5 or 6, depending on the size of the ulcers. Consequently, CLTI with arterial leg ulcers represents the most advanced form of PAD [4, 5], putting patients subsequently at higher risk of dying from infection related causes. Despite its importance, evidence for empirical antibiotic treatment of infected arterial leg ulcers in people with CLTI remains scarce and guideline-based recommendations are not currently available [6, 7].

In addition to revascularisation, antibiotic therapy is often used as supportive therapy to aid wound healing in the event of infection. In particular, empirical antibiotic treatment is often used in PAD patients for several reasons. Firstly, PAD, especially CLTI, is a chronic disease with frequent use of outpatient services, where empiric treatment is a well-established therapeutic strategy. Secondly, in critically ill patients, especially prior to revascularisation, empirical therapy is crucial and often used to prevent systemic dissemination [8, 9]. Infection-related indications are the most common reason for 30-day readmission after endovascular revascularisation in patients with CLTI, accounting for more than forty percent [8]. This highlights the importance of appropriate antibiotic therapy to prevent systemic inflammation in PAD patients following revascularisation.

To be effective, empirical treatment regimens must be evidence-based and well-studied. Empirical therapy of infected ulcers in patients with diabetic foot infections (DFI) is a prime example [10]. However, the applicability of these DFI guidelines to arterial leg ulcers is highly questionable and not well investigated. Notable recent study have demonstrated the impact of coexisting PAD in patients with DFI as an independent risk factor for non-healing wounds [11].

The present study aims to provide insight into the microbiological patterns and antimicrobial resistance (AMR) of specific pathogens in patients with arterial leg ulcers. Second, to identify independent risk factors for Staphylococcus aureus-associated foot infections in patients with PAD using logistic regression.

## Methods

### Study design

We retrospectively analysed data derived from a consecutive cohort of endovascular interventions in patients with PAD Rutherford category 5 or 6 performed at the Department of Cardiology and Angiology, University Heart Center Freiburg-Bad Krozingen, Germany, from 2012 to 2021. The data included microbiological diagnostics of infected wound specimens from all enrolled patients. Microbiological diagnostics and data storage were performed by Dr. Haas, Dr. Raif and colleagues at the medical laboratories of the private laboratory group Medical Care Center (MVZ) Clotten. The microbiological data sets were extracted using the hygiene management system HyBASE and stored as pseudonymized Excel files.

### Study participants

**Fig 1:**
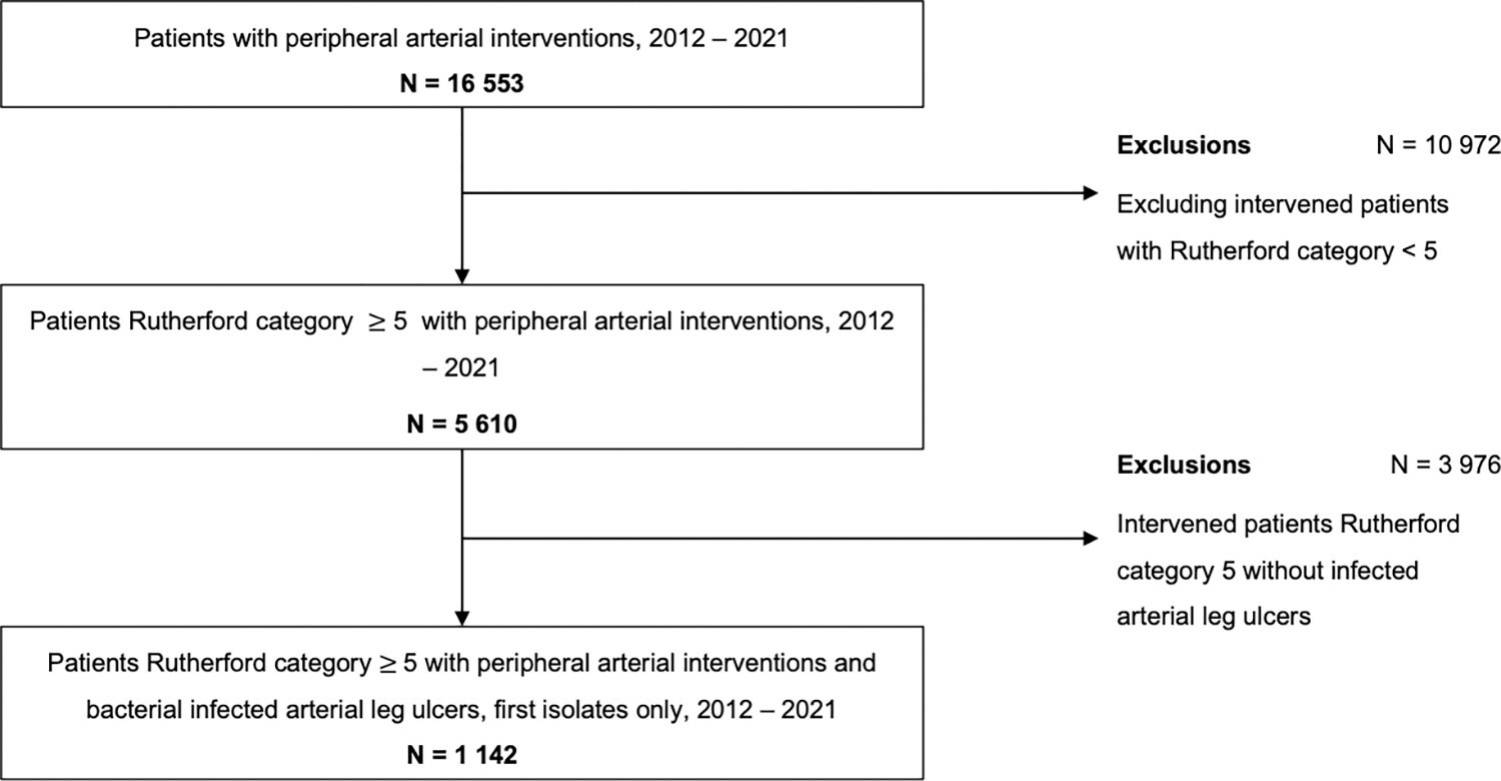
Study populations inclusion and exclusion criteria from 2012 to 2021.

All patients with infected arterial leg ulcers and angiographically confirmed peripheral occlusive disease of Rutherford category 5 and 6 were included in the study (Fig 1). On admission, patients were graded by physicians according to Rutherford categories. Next, all patients graded Rutherford category 5 or 6 received wound care by a trained wound care specialist. Meanwhile, the wound care specialists assessed the wounds macroscopically for signs of infection. Only wounds with local signs of infection were sampled. If judgement was difficult, physicians were consulted and the wound was reevaluated. For every study participant we had complete microbiological test results of their corresponding wound specimens. Since one patient could be infected with more than one bacterium, there are fewer patients than bacterial isolates. The Rutherford classification stratifies the disease into six categories according to the principle that the higher the number the worse the disease presents. Patients are classified according to the severity of symptoms (i.e. claudication) and non-invasive objective criteria (i.e. treadmill test) [4].

### Ethics

Approval for the study was given by the Ethical Board of the Universität Freiburg (proposal-number: 22-1238-S1-retro). The need for participant informed consent was waived by the ethics committee.

### Specimens and microbiology

Wound specimens and wound swabs were collected by a trained wound manager following a standardized protocol as follows: 1. Wound cleaning and disinfection with Octenispet (Schülke, Noderstedt, Germany) or Granudacyn (Mölnlycke, Gothenburg, Sweden), aqueous wound and mucous membrane antiseptics; 2. Wound debridement via mechanical or autolytical abrasion of avital tissue; 3. Tissue scrape from the ulcer base via sterile scalpel or deep wound specimens if collection is not possible. Pathogen identification and antimicrobial susceptibility testing (AST) were performed using automated systems such as MALDI-TOF, Vitek2, disc diffusion and microbroth dilution. We included susceptibility testing to: Oxacillin (OXA); Ampicillin (AMP); Amoxicillin/clavulanic acid (AMC); Piperacillin (PIP); Piperacillin/tazobactam (TZP); Ceftazidime (CAZ); Cefepime (FEP); Meropenem (MEM), Clindamycin (CLN); Ciprofloxacin (CIP); Trimethoprim/sulfamethoxazole (SXT) and Vancomycin (VAN). The results were interpreted according to the European Committee on Antimicrobial Susceptibility Testing (EUCAST) [12] and the German National Committee for sensitivity testing of antibiotics (Nationales Antibiotika-Sensitivitätstest-Komitee; NAK) [13]. To minimize overestimation of microbiological isolates due to multiple identification within the same individual patient, we only included first isolates per phenotype according to Hindler et al. as implemented in the R package AMR [14, 15]. Multi-drug resistance (MDR) was defined according to the definition by Magiorakos et al. [16]. This definition was proposed by a joint initiative of the European Centre for Disease Prevention and Control and the Centers for Disease Control and Prevention. Accordingly, MDR was defined as acquired nonsusceptibility to at least one agent in three or more antimicrobial categories, whereas extensively-drug resistance (XDR) was defined as nonsusceptibility to at least one agent in all but two or fewer antimicrobial categories [16].

### Statistical analysis

Descriptive analyses for patient characteristics are reported as means with standard deviation (SD) for continuous variables and as counts with percentages for categorical variables. Antimicrobial susceptibility of a species is reported as the percentage of resistant isolates among all isolates tested. We calculated 95% confidence intervals (CI) for proportions using the Clopper–Pearson method. Logistic regression was performed to identify risk factors for *S*. *aureus*-associated wound infections. The selection of predictor variables was done a priori using subject matter knowledge. The results of the logistic regression models are reported as odds ratios (OR) with 95% CIs and corresponding p values. The evaluation of the AST per pathogen as well as logistic regression modelling were performed as complete case analyses. All statistical analyses were performed using the free statistical computing and graphics software R (R 4.0.3; R Foundation, Vienna, Austria). The significance level was set to α  =  .05.

## Results

### Study sample

A total of 28,994 peripheral arterial interventions were performed in 16,553 individual patients between 2012 and 2021. After applying all inclusion and exclusion criteria, a total of 1,142 patients were included in the study (Fig 1). The study population’s mean age was 75.6 (SD: ± 10.9) years with a higher prevalence in males (64.7%). Nicotine consumption is the least common cardiovascular risk factor in patients with PAD Rutherford category 5 and 6 (40.7%). The median number of arteries treated was 2 (Table 1).

**Table 1:** Patient characteristics.

	Overall (N = 1 142)
**Demographics**	
Age–y mean (SD)	75.6 (10.9)
Sex–female n (%)	404 (35.4)
**Comorbidities** (%)	
Arterial hypertension	1 042 (91.6)
Hypercholesterolemia	882 (77.6)
Diabetes mellitus	695 (61.1)
Nicotine consumption	463 (40.7)
End-stage renal disease	113 (9.9)
**Intervention / Infection Site (%)**	
Right	565 (49.5)
Left	531 (46.5)
Bilateral	45 (3.9)
**Emergency (%)**	
Urgent intervention (<24h)	87 (7.6)
**Intervened vessels (%)**	
Infrapopliteal arteries*	805 (70.5)
Popliteal artery	599 (52.5)
Superficial femoral artery	548 (48.0)
Common femoral artery	91 (8.0)
External iliac artery	95 (8.3)
Profunda femoris artery	65 (5.7)
Common iliac artery	59 (5.2)
**Number of treated arteries**	
Median (IQR)	2 (2.0, 3.0)

* Infrapopliteal arteries: Tibioperoneal trunk, anterior tibial artery, posterior tibial artery, fibular artery

### Microbiological patterns

A total of 2,335 isolates (68.1%) were gram-positive, while 1,096 isolates (31.9%) were gram-negative (Table 2). The most prevalent bacteria are *S*. *aureus* (18.6%), *Enterococcus faecalis* (9.1%) and *S*. *epidermidis* (7.8%). The most common gram-negative bacteria are *Pseudomonas aeruginosa* (5.6%), *Proteus mirabilis* (3.7%) and *Escherichia coli* (3.4%). There is no discernible difference in microbiological patterns in PAD patients with or without coexisting diabetes mellitus (Table 2).

**Table 2:** Frequency of pathogens isolated from arterial leg ulcers in patients with peripheral artery disease stratified by diabetes mellitus, category Rutherford 5 or 6, 2012 to 2021.

	Overall^†^ (N = 3 431)	Non-Diabetes mellitus (N = 1 294)	Diabetes mellitus (N = 2 112)
**Polymicrobial n (%)**	2,912 (84.9)	1,119 (86.5)	1,770 (83.8)
**Pathogen n (%)**			
**Gram positive (n = 2335**^**§**^**)**			
*Staphylococcus aureus*	638 (18.6)	243 (18.8)	392 (18.6)
*Enterococcus faecalis*	311 (9.1)	111 (8.6)	197 (9.3)
*Staphylococcus epidermidis*	268 (7.8)	93 (7.2)	175 (8.3)
*Dermabacter hominis*	110 (3.2)	37 (2.9)	73 (3.5)
*Streptococcus group B*	81 (2.4)	22 (1.7)	57 (2.7)
*Staphylococcus lugdunensis*	74 (2.2)	34 (2.6)	40 (1.9)
*Corynebacterium amycolatum*	65 (1.9)	27 (2.1)	38 (1.8)
*Corynebacterium striatum*	65 (1.9)	23 (1.8)	42 (2.0)
*Staphylococcus caprae*	63 (1.8)	17 (1.3)	46 (2.2)
*Staphylococcus capitis*	56 (1.6)	25 (1.9)	31 (1.5)
*Staphylococcus haemolyticus*	54 (1.6)	24 (1.9)	30 (1.4)
*Corynebacterium tuberculostearicum*	38 (1.1)	13 (1.0)	25 (1.2)
**Gram negative (n = 1096**^**§**^**)**			
*Pseudomonas aeruginosa*	193 (5.6)	82 (6.3)	110 (5.2)
*Proteus mirabilis*	126 (3.7)	46 (3.6)	79 (3.7)
*Escherichia coli*	115 (3.4)	47 (3.6)	68 (3.2)
*Enterobacter cloacae*	106 (3.1)	34 (2.6)	71 (3.4)
*Klebsiella oxytoca*	73 (2.1)	27 (2.1)	44 (2.1)
*Stenotrophomonas maltophilia*	71 (2.1)	28 (2.2)	42 (2.0)
*Morganelle morganii*	49 (1.4)	20 (1.5)	28 (1.3)
*Proteus vulgaris*	36 (1.0)	14 (1.1)	22 (1.0)
*Serratia marcescens*	36 (1.0)	15 (1.2)	21 (1.0)
*Klebsiella pneumoniae*	35 (1.0)	8 (0.6)	27 (1.3)
** Obligate anaerobic**			
*Finegoldia magna*	69 (2.0)	27 (2.1)	41 (1.9)
Others*	699 (20.4)	277 (21.4)	413 (19.6)

* Every bacteria with a frequency below 1%

^†^ Polymicrobial infections: n = 2,912

^$^ Only bacteria with a frequency greater than 1% are listed. Therefore, the sum of the bacteria listed is lower than the total number

### Antimicrobial susceptibility

**Fig 2:**
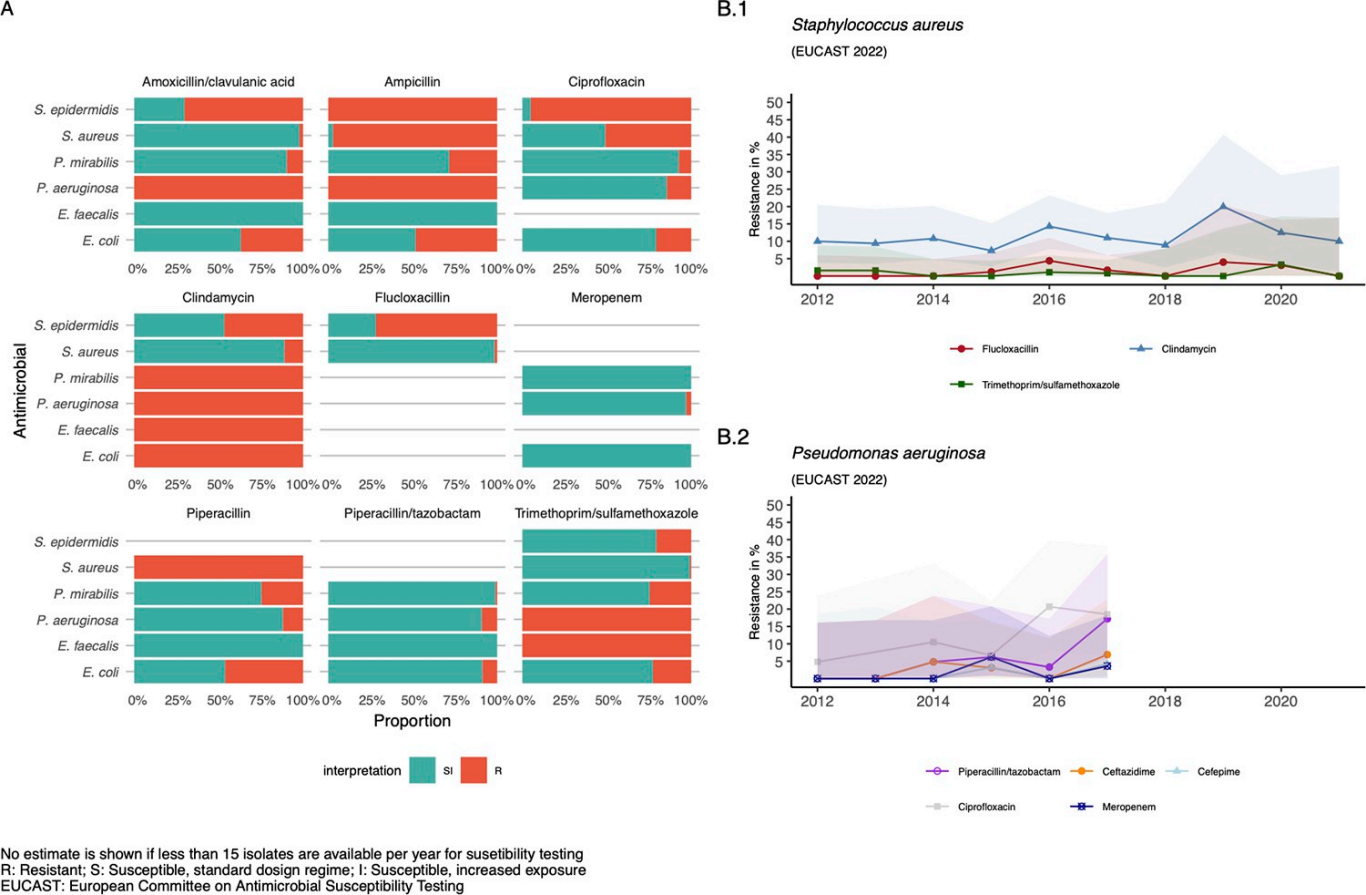
Antimicrobial resistance per bacterial isolate detected in patients with peripheral artery disease Rutherford category 5 and 6 as point estimate and over time, 2012 to 2021. A) Proportion of susceptible isolates among all isolates detected in patients with PAD and ischaemic wounds. No estimate is given if less than 15 isolates per year are available for susceptibility testing, green bars represent susceptibility, red bars indicate resistance. (B.1) Antimicrobial resistance of *S*. *aureus* to flucloxacillin, clindamycin and trimethoprim/sulfamethoxazole over time, 2012–2021. (B.2) Antimicrobial resistance of *P*. *aeruginosa* to piperacillin/tazobactam, ceftazidime, cefepime, ciprofloxacin and meropenem over time, 2012–2021.

Resistance of *S*. *aureus* isolates to CLN was frequent (11.0%) whereas resistance to OXA remains scarce (1.5%). No VAN resistant *E*. *faecalis* isolates were detected from PAD patients, whereas 0.12% VAN resistant *E*. *faecalis* isolates and 5.6% OXA resistant *S*. *aureus* isolates were detected during this study period regardless of sample origin (S1 and S2 Tables in S1 File). *P*. *aeruginosa*, had a high resistance to CIP (14.4%) but resistance to CAZ and MEM remains scarce (4,8%; 2,9%). *E*. coli was frequently resistant to AMC, CIP and SXT (36.8%; 20.7%; 22.6%), respectively. In contrast resistance to CAZ was rare (3.6%) and no MEM resistant *E*. *coli* isolates were detected (Table 3; Fig 2A).

**Table 3:** Antimicrobial resistance of bacteria isolated from arterial leg ulcers in patients with peripheral artery disease Rutherford category 5 and 6, 2012–2021.

	*S*. *aureus* (N = 638)		*E*. *faecalis* (N = 311)		*S*. *epidermidis* (N = 268)		*P*. *aeruginosa* (N = 193)		*P*. *mirabilis* (N = 126)		*E*. *coli* (N = 115)	
	R (%)	n	R (%)	n	R (%)	n	R (%)	n	R (%)	n	R (%)	n
**Antibiotics**												
OXA	1.5	609	ND	0	71.8	131	ND	0	ND	0	ND	0
AMP	97.1	382	0.0	291	100.0	95	-*	193	28.3	99	48.2	110
AMC	2.1	421	0.0	291	70.2	104	-*	193	9.6	94	36.8	106
PIP	100.0	371	0.0	291	ND	0	11.9	168	24.6	114	46.1	115
TZP	NA	0	0.0	291	ND	0	9.0	167	0.9	111	8.7	104
CAZ	-*	638	-*	311	ND	0	4.8	167	0.0	112	3.6	111
FEP	ND	0	-*	311	ND	0	2.6	152	ND	0	-^†^	2
MEM	ND	0	ND	0	ND	0	2.9	174	0.0	114	0.0	115
CLN	11.0	611	-*	311	46.6	131	-*	193	-*	126	-*	115
CIP	50.8	193	-^†^	13	95.0	80	14.4	153	7.3	110	20.7	111
SXT	0.8	610	-*	265	20.6	131	-*	178	24.6	114	22.6	115
VAN	0.2	611	0.0	292	0.0	131	-*	193	-*	126	-*	115

OXA: Oxacillin; AMP: Ampicillin; AMC: Amoxicillin/clavulanic acid; PIP: Piperacillin; TZP: Piperacillin/tazobactam; CAZ: Ceftazidime; FEP: Cefepime; MEM: Meropenem, CLN: Clindamycin; CIP: Ciprofloxacin; SXT: Trimethoprim/sulfamethoxazole; VAN: Vancomycin

R: Resistance in percent; n: Number of tested isolates

-*: intrinsically resistant

-^†^: Not enough tested isolates for reliable estimates, ND: Not done

CLN resistance among *S*. *aureus* isolates is increasing over time, whereas OXA and SXT resistance are constant over time at a low rate. TZP and CIP resistance in *P*. *aeruginosa* is increasing over time (Fig 2B).

**Fig 3:**
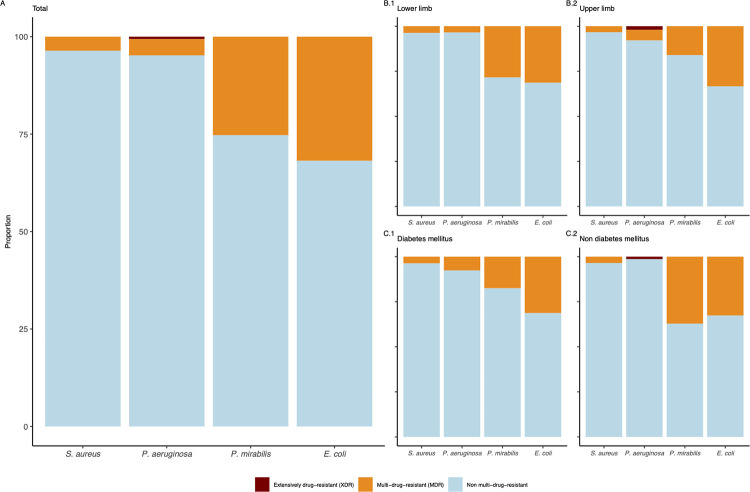
Multi-drug resistance according to Magiorakos et al. in bacteria isolated from patients with peripheral artery disease Rutherford category 5 and 6, stratified by site of intervention and diabetes status, 2012 to 2021. (A) Bacterial isolates detected in ischaemic ulcers that are multi- or extensively drug resistant according to Magiorakos et al. in total (A) and stratified by PAD site (B.1 and B.2) as well as diabetes status (C.1 and C.2).

MDR is rare in *S*. *aureus* and *P*. *aeruginosa* isolates. E. coli isolates have the highest proportion of MDR of all species. XDR can only be detected in *P*. *aeruginosa* isolates (Fig 3A). When stratifying by site of PAD (lower limb versus upper limb, Fig 3B), there are no major changes in this pattern. However, stratification by diabetes status (Fig 3C) shows higher rates of MDR in *P*. *mirabillis* and *E*. *coli* isolates identified in patients without diabetes.

### Staphylococcus aureus

As the most common pathogen detected in ischaemic wounds, *S*. *aureus* appears to play an important role in wound infection in PAD. Therefore, we performed a multivariable logistic regression model to identify risk factors *for S*. *aureus* detection in wound infections in patients with PAD Rutherford category 5 and 6 (Table 4). *S*. *aureus* identification was independently positively associated with end-stage renal disease (p = .042) (Table 4).

**Table 4:** Risk factors for *S*. *aureus* identification in arterial leg ulcers in patients with peripheral artery disease Rutherford category 5 and 6.

Variable / Category*	OR	95% CI	p value
Sex			
Male	Reference		
Female	0.887	0.681; 1.155	.37
**Nicotine consumption**			
Non Nicotine consumption	Reference		
Nicotine consumption	1.137	0.882; 1.467	.32
**Renal disease**			
Non Dialysis	Reference		
End-stage	1.517	1.019; 2.278	.042
**Treated Lesion**			
Femoropopliteal	Reference		
Infrapopliteal	0.852	0.648; 1.118	.25
**Glucose metabolism**			
Non-Diabetes	Reference		
Diabetes	1.037	0.807; 1.333	.77
**Cholesterol**			
Non elevated	Reference		
Hypercholesterolemia	0.868	0.651; 1.156	.33

OR: Odds Ratio

95% CI: 95% confidence interval

* Logistic regression analysis is based on all presented variables

## Discussion

This study evaluated clinical characteristics and microbiological test results (n = 3431) in leg ulcers from a large cohort of PAD patients with Rutherford category 5 and 6 between the years 2012 and 2021 (n = 1,142). *S*. *aureus* was identified as the most prevalent pathogen (19%) and CLN resistance was common (11%).

Evidence regarding infected arterial leg ulcers is scarce and empirical therapy recommendations are often transferred from the clinical practice guideline for the diagnosis and treatment of diabetic foot infections by the Infectious Diseases Society of America [10]. Nevertheless, based on our results, the microbiological pattern in patients with PAD, regardless of their diabetic status, differs from that reported for diabetic patients alone. In both groups gram positive bacteria are the most commonly isolated. However, in DFI higher frequencies of gram-positive bacteria are reported with approximately 80% compared to 68% in the present findings [17, 18]. Despite the lack of significant differences in microbiological patterns, there are differences in clinical outcomes between diabetic and PAD patients. This can be seen in the reasons for non-healing wounds. Recent studies have identified wound-infection as an independent risk factor for non-healing only in patients with diabetes and PAD–not in diabetic patients without concomitant PAD [11]. Taken together, this emphasizes the importance of further investigating the differences in patients with diabetes alone and those with PAD with or without concomitant diabetes. Interestingly, we could not identify any differences in microbiological patterns in PAD patients with or without coexisting diabetes mellitus.

CLN is commonly used to treat skin and soft tissue infections (SSTIs), as documented in a recent cohort study of outpatient veterans diagnosed with SSTIs in which 40% were prescribed anti-MRSA antibiotics (clindamycin, daptomycin, linezolid, sulfonamides, tetracyclines, tigecycline, vancomycin).[19] Despite its frequent use in SSTI infections, CLN cannot be recommended as an empirical drug for patients with infected arterial leg ulcers due to low empirical susceptibility. The prominent role of CLN as an antibiotic in arterial leg ulcers may be partly explained by an adequate tissue penetration due to its oral bioavailability [20]. Nevertheless, orally administered CLN has serious adverse effects such as pseudomembranous colitis with an OR of 17 compared to non-antibiotic exposure [21]. Based on our results, we suggest AMC as a potential empirical therapy option for arterial leg ulcers with a high empirical susceptibility. Recent study confirm adequate tissue penetration, which is not inferior to clindamycin [22], further supports our recommendation.

The role of coagulase-negative staphylococci (CoNS) such as *S*. *epidermidis* in skin and soft tissue infections is important to note. CoNS are common commensals of the skin. As SSTIs are often polymicrobial in nature, it can be difficult to distinguishing between contaminants and causative pathogens. Undoubtedly, CoNS can be causative in monomicrobial infections. In general, the pathogenicity of CoNS is thought to be lower than that of *S*. *aureus*. However, their pathogenicity is impressively demonstrated by the fact that CoNS are the leading cause of nosocomial bloodstream infections (BSI) in US hospitals [23]. That being said, the role of CoNS in polymicrobial ischaemic leg ulcers is unclear and further, prospective, studies are needed to clarify this.

According to the present findings, smoking, regardless if former or current, was the least frequent risk factor of a set of typical cardiovascular risk factors. These findings are consistent with previous findings [24, 25]. Furthermore, when risk factors are stratified by disease severity according to Rutherford categories, the frequency of smoking as a risk factor declines significantly [24].

In the present findings, we identified low rates of VAN resistant *E*. *faecalis* (0%) and OXA resistant *S*. *aureus* (1.5%) in ischaemic wounds. During the study period, higher frequencies were found in overall hospital specimens with 0,12% and 5.6% respectively. Importantly, these hospital-wide resistance rates were calculated irrespective of the sample origin. This indicates differences in resistance patterns depending on the origin of the sample. However, This difference remains unexplained and must be interpreted with caution.

Recent studies have identified hemodialysis as an independent risk factor for *S*. *aureus* bloodstream infections, with vascular catheters being the vascular access with the highest risk [26, 27]. These findings are consistent with the recent evidence that end-stage renal disease is an independent risk factor for the identification of *S*. *aureus* in ischaemic leg ulcers.

The present study has several limitations. First, its retrospective design introduces an inherent risk of bias. Second, single-centre studies often lack external generalizability, especially in microbiological studies, due to potential regional differences in pathogen stratifications. Nevertheless, we consider the latter to be a less pronounced bias in this study due to the large hospital catchment area, including the whole of Germany with a focus on Baden-Württemberg. Furthermore, we did not include laboratory parameters or vital signs related to infection parameters such as C-reactive protein or body temperature. Not including these variables could bias the estimates towards higher proportions of commensals rather than causative pathogens. Finally, as our analysis are based on the final microbiological test results, we cannot exclude the possibility of pre-selection of pathogens in terms of suppression of potential commensals or irrelevant pathogens by clinical microbiologists.

## Conclusion

*S*. *aureus* is the most common pathogen in arterial leg ulcers, and end-stage renal disease is an independent risk factor. Arterial leg ulcers in patients with and without coexisting Diabetes mellitus do not differ in their microbiological patterns. As clindamycin resistance of *S aureus* isolates is common, we conclude that its use as an empirical agent is highly questionable. The high proportion of MDR in *E*. *coli* isolates is of concern and the non-essential use of antibiotics in PAD patients should be limited.

## Supporting information

S1 File Frequency of vancomycin-resistant *Enteroccoci* (VRE) stratified by species and frequency of methicillin-resistant *Staphylococcus aureus* (MRSA), University heart center Bad Krozingen, 2010 to 2021. (DOCX)
