# Supplementary material for: Arterial leg ulcers—Bacterial patterns, antimicrobial resistance and clinical characteristics, a retrospective single-centre cohort, 2012–2021
Source: PLoS One. 2023 Aug 11;18(8):e0290103. doi: 10.1371/journal.pone.0290103 (PMC10420368; doi:10.1371/journal.pone.0290103)
Supplement: S1 File — (DOCX) [file pone.0290103.s001.docx]

| **S1 Table:** Frequency of vancomycin-resistant *Enteroccoci* (VRE) stratified by species, University heart center Bad Krozingen, 2010 to 2021 | | | | | | | | | |
| --- | --- | --- | --- | --- | --- | --- | --- | --- | --- |
|  | **Total** | | | ***Enterococcus faecalis*** | | | ***Enterococcus faecium*** | | |
|  | **VRE**  **n** | **Total**  **n** | **% (95% CI)** | **VRE**  **n** | **Total**  **n** | **% (95% CI)** | **VRE**  **n** | **Total**  **n** | **% (95% CI)** |
| Total | 77 | 2008 | 3.83 (3.08 - 4.77) | 2 | 1661 | 0.12 (0.03 - 0.48) | 75 | 347 | 21.61 (17.58 - 26.28) |
| **Material** | | | |  |  |  |  |  |  |
| Wound swaps | 39 | 1476 | 2.64 (1.94 - 3.60) | 1 | 1289 | 0.08 (0.01 - 0.55) | 38 | 187 | 20.32 (15.13 - 26.74) |
| Urine | 8 | 179 | 4.47 (2.25 - 8.70) | 0 | 124 | 0 (NA) | 8 | 55 | 14.55 (7.38 - 26.67) |
| Blood | 3 | 79 | 3.80 (1.22 - 11.20) | 0 | 62 | 0 (NA) | 3 | 17 | 17.65 (5.57 - 43.77) |
| Heart valves^1^ | 1 | 14 | 7.14 (0.92 - 38.87) | 0 | 13 | 0 (NA) | 1 | 1 | 100.00 (NA) |
| Others^2^ | 26 | 260 | 10.00 (6.89 - 14.29) | 1 | 173 | 0.58 (0.08 - 4.01) | 25 | 87 | 28.74 (20.15 - 39.18) |
| **Type of care** | | | |  |  |  |  |  |  |
| Normal care | 33 | 1262 | 2.61 (1.86 - 3.66) | 1 | 1086 | 0.09 (0.01 - 0.65) | 32 | 176 | 18.18 (13.13 - 24.63) |
| Intensive care | 40 | 481 | 8.32 (6.16 - 11.15) | 0 | 311 | 0 (NA) | 40 | 159 | 25.16 (18.99 - 32.53) |
| Outpatient | 4 | 265 | 1.51 (0.57 - 3.96) | 1 | 253 | 0.40 (0.06 - 2.76) | 3 | 12 | 25.00 (7.81 - 56.73) |
| NA: Not available; CI: Confidence interval  ^1^ Tissue from heart valves (i.e. mitral valve, aortic valve)  ^2^ Bronchoalveolar lavage; excision; pleural punctate; unspecified; nasal swap; punctate unspecified; pharyngeal swap; sputum | | | | | | | | | |

| **S2 Table:** Frequency of methicillin-resistant *Staphylococcus aureus* (MRSA), University heart center Bad Krozingen, 2010 to 2021 | | | |
| --- | --- | --- | --- |
|  | **MRSA n** | **Total n** | **% (95% CI)** |
| Total^1^ | 195 | 3475 | 5.61 (4.89 - 6.43) |
| **Material** | | | |
| Wound swaps | 123 | 2866 | 4.29 (3.61 - 5.10) |
| Blood | 11 | 192 | 5.73 (3.20 - 10.06) |
| Heart valves^2^ | 0 | 18 | 0 (NA) |
| Urine | 0 | 14 | 0 (NA) |
| Others^3^ | 61 | 385 | 15.84 (12.52 - 19.85) |
| **Type of care** | | | |
| Normal care | 128 | 2279 | 5.62 (4.74 - 6.64) |
| Intensive care | 38 | 448 | 8.48 (6.23 - 11.45) |
| Outpatient | 29 | 748 | 3.88 (2.71 - 5.53) |
| NA: Not available; CI: Confidence interval  ^1^ No routine swaps included  ^2^ Tissue from heart valves (i.e. mitral valve, aortic valve)  ^3^ Bronchoalveolar lavage; excision; pleural punctate; unspecified; punctate unspecified; sputum | | | |
